# High inhaled oxygen concentration quadruples exhaled CO in healthy volunteers monitored by a highly sensitive laser spectrometer

**DOI:** 10.1038/s41598-019-48789-8

**Published:** 2019-08-22

**Authors:** Vivien Brenckmann, Irène Ventrillard, Daniele Romanini, Kévin Jaulin, Pascale Calabrèse, Raphaël Briot

**Affiliations:** 1 Emergency Department, Grenoble Alpes University Hospital, Grenoble, France; 2 Laboratory TIMC; Team PRETA; UMR 5525 CNRS - University Grenoble Alpes, Grenoble, France; 3 Laboratory LIPhy, UMR 5588 CNRS - University Grenoble Alpes, Grenoble, France; 4 AP2E company, Aix-en-Provence, France

**Keywords:** Translational research, Respiration, Laser material processing

## Abstract

Carbon monoxide (CO) monitoring in human breath is the focus of many investigations as CO could possibly be used as a marker of various diseases. Detecting CO in human breath remains a challenge because low concentrations (<ppm) must be selectively detected and short response time resolution is needed to detect the end expiratory values reflecting actual alveolar concentrations. A laser spectroscopy based instrument was developed (ProCeas) that fulfils these requirements. The aim of this study was to validate the use of a ProCeas for human breath analysis in order to measure the changes of endogenous exhaled CO (eCO) induced by different inspired fractions of oxygen (FiO_2_) ranging between 21% and 100%. This study was performed on healthy volunteers. 30 healthy awaked volunteers (including asymptomatic smokers) breathed spontaneously through a facial mask connected to the respiratory circuit of an anesthesiology station. FiO_2_ was fixed to 21%, 50% and 100% for periods of 5 minutes. CO concentrations were continuously monitored throughout the experiment with a ProCeas connected to the airway circuit. The respiratory cycles being resolved, eCO concentration is defined by the difference between the value at the end of the exhalation phase and the level during inhalation phase. Inhalation of 100% FiO_2_ increased eCO levels by a factor of four in every subjects (smokers and non smokers). eCO returned in a few minutes to the initial value when FiO_2_ was switched back to 21%. This magnification of eCO at 21% and 100% FiO_2_ is greater than those described in previous publications. We hypothesize that these results can be explained by the healthy status of our subjects (with low basal levels of eCO) and also by the better measurement precision of ProCeas.

## Introduction

Monitoring the endogenous carbon monoxide in exhaled breath (eCO) is of growing interest in clinical studies. This rapid and non-invasive measurement may reveal various pathological conditions.

Carbon monoxyde (CO) has been extensively studied, as it is well known as a toxic gas in ambient air due to incomplete combustion of organic products. Beyond this external toxic effect, CO is normally produced endogenously by the degradation of heme porphyrins to biliverdin and iron (bilirubin metabolism or heme catbolism). This reaction is catalyzed by the rate-limiting enzyme family of heme oxygenases (HO). Several sources of stress including inflammation and ischemia-reperfusion injury, dramatically increased expression of HO-1, the inducible isoform, leading to increased levels of endogenous CO^1^. Thus endogenous CO is not the cause of the diseases but it could be an interesting marker to monitor closely. Endogenous CO is eliminated from the body in exhaled air *via* the normal respiratory process. CO concentration in exhaled air (*i.e*. eCO) is thus a good indicator of the actual values of this molecule in the body. The eCO level increases in several airways inflammatory diseases like asthma^2^, COPD^3^, and allergic rhinitis^4^. eCO is also increased in some systemic diseases such as severe sepsis occurring in cirrhotic patients^5^. Moreover it is a predictive marker of obliterative bronchiolitis which occurs after lung transplantation^6^.

The increase in CO production, in turn, exerts anti-inflammatory, anti-apoptotic and anti-proliferative effects and limits ischemia-reperfusion injury consequences. For example, reduction of CO production (by inhibition of HO enzyme activity) after experimental lung ischemia-reperfusion, increases alveolar cell damage, recruitment of inflammatory cells, and lung edema. In contrast, inhalation of small concentrations of exogenous CO (100 ppm to 500 ppm), reduces apoptosis, excretion of inflammatory mediators and pulmonary edema caused by ischemia-reperfusion injury^1^.

ECO concentrations in healthy subjects reach 1.5 ppm up to more than 10 ppm depending on smoking status of the subjects^7^. Some clinical studies require eCO continuous monitoring with a sensitive and reliable device allowing cycle to cycle variation measurements by high frequency sampling. This allows to accurately monitor the eCO deduced from the value measured at the end of the exhalation phase to which is subtracted the CO background measured during the inhalation phase. This background corresponds to CO concentration in inhaled gas that may be subject to change. As an example, in a clinical room, CO concentration in ambient air can vary daily in a typical range of 0.3 to 0.9 ppm. In medical gas supplies (medical air or oxygen) CO concentration is usually lower (<0.2 ppm) and more stable^8^. The recently developped laser spectroscopy technique named OF-CEAS (for Optical Feedback - Cavity Enhanced Absorption Spectroscopy), has been described in detail in previous publications^8–10^. The instrument implementing this technique, commercialized by the French company AP2E, goes under the denomination of ProCeas used further below. The OF-CEAS technique has the advantage, relative to others laser spectroscopy methods^11^ to offer an exceptionally low CO detection limit (typically 1 part per billion: 1 ppb) over a short response time (about 1 second) and using a modest gas flow (0.15 L/min). Moreover, OF-CEAS provides real time measurements without the need for a periodic calibration. The instrument is compact and can be operated by non-specialists in a medical environment.

Since endogenous CO is rather slowly eliminated in exhaled air, and its elimination is accelerated by a high oxygen rate, high oxygen concentrations are clinically used as a treatment in severe CO poisoning. Hyperbaric oxygen therapy is broadly used for carbon monoxide poisoning although its efficacy and details of implementation remain controversial^12,13^. Increasing FiO2 was already used for studying variations of eCO in spontaneously breathing patients just before cardiac surgery^14^ and in mechanically ventilated patients under general anesthesia^15,16^.

The aim of this study is to test the relevance of the OF-CEAS technique to monitor eCO variations due to modified FiO_2_ in healthy volunteers. Additionally, we compared ProCeas real-time measurements with values obtained by sample analysis of exhaled gas with a standard electrochemical handheld sensor (MicroCO; VYaire).

## Methods

A prospective monocentric clinical trial in adult healthy volunteers was conducted over a one-month period. The protocol was carried out in accordance with relevant guidelines and regulations of French law concerning persons participating in biomedical research. The study protocol was registered in ClinicalTrials.gov (ClinicalTrials.gov identifier: NCT01881945) and was approved by the French “Comity for Protection of People” (CPP of region South East 5) (registration number 2008-A00273-52). Thirty healthy volunteers participated to the experiment. Informed consent was obtained from each subject. For medical gas supply convenience, measurements took place in an operating room of the Grenoble University hospital. The healthy subjects, comfortably seated and perfectly awake, would breath at rest through a facial mask connected to the airway circuit of an anesthesiology station (Primus Dräger). This station was connected to the regular medical gas supply (air and oxygen) of the operating room. Once the subject was installed, he/she was required to hold the mask tightly against his/her face and asked to breath through the mask at a resting rate. Initially the mask was only connected to an antiparticule filter, so that the subject would breath ambient air. Then the mask and the filter were connected to the tubing circuit of the anesthesiology station. The airway circuit of the station was set in manual mode for spontaneous breathing (*i.e*. no mechanical ventilation) and on “open circuit” with a fresh gas flow rate sufficient to prevent rebreathing.

Different values of the inspired fraction of oxygen were applied through the control panel of the anesthesiology station. Over a period of 30 minutes, eCO levels were recorded during different steps (ambient air, FiO_2_ 21%, FiO_2_ 50%, FiO_2_ 100%). Each step had a five minute duration of steady-state duration.

### eCO measurement by optical feedback cavity-enhanced absorption spectroscopy (OF-CEAS)

**Figure 1:**
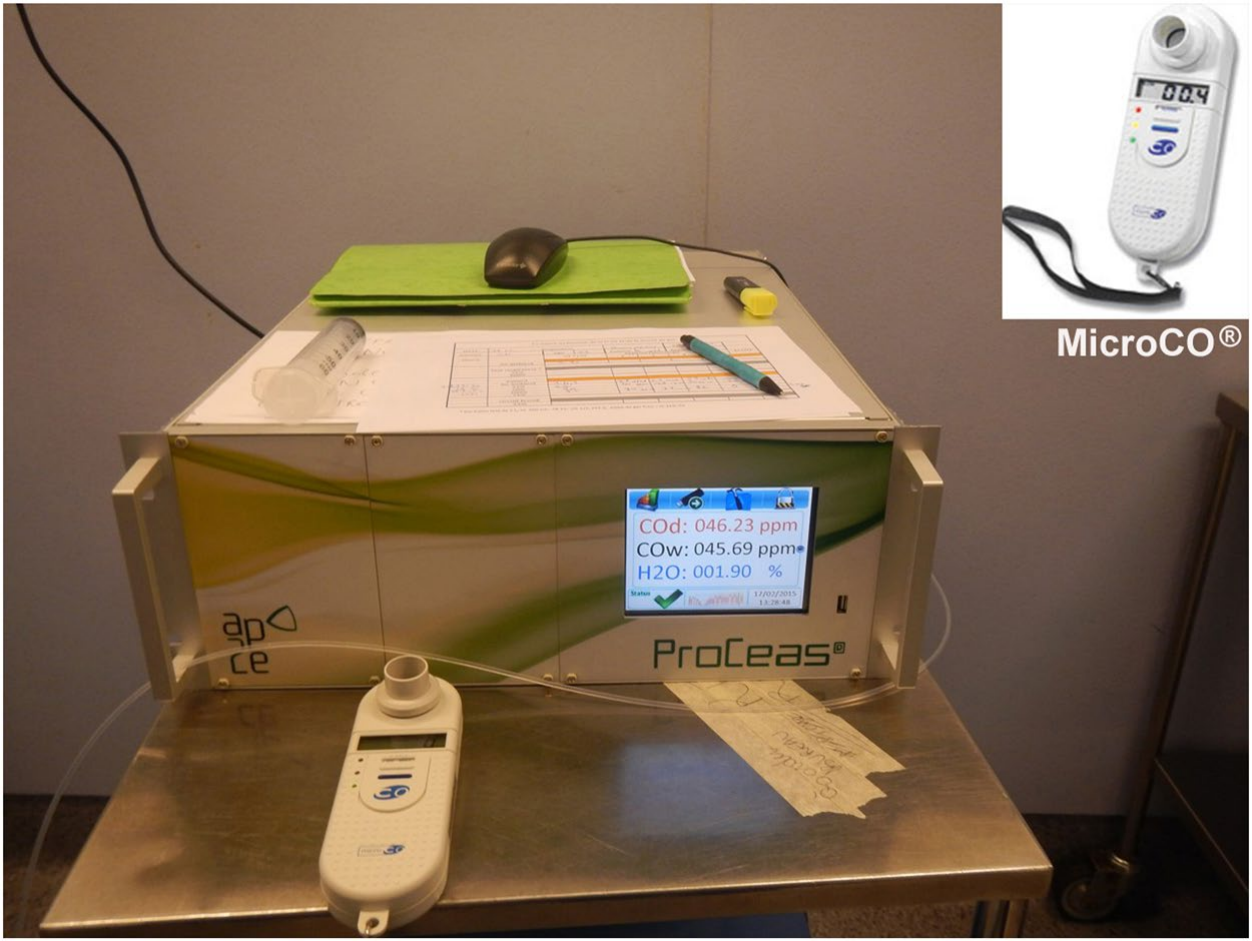
eCO was measured by both ProCeas and MicroCO devices.

**Figure 2:**
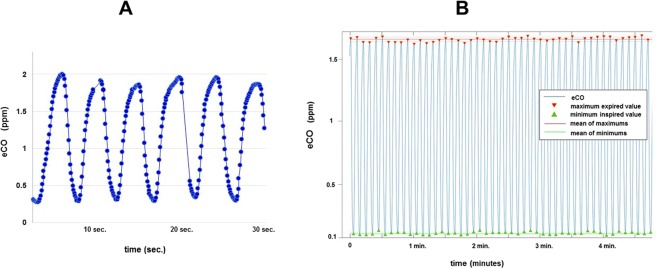
(**A,B**) eCO curves recorded with a ProCeas for a non-smoker healthy volunteer breathing medical air (21% FiO_2_). (**A**) eCO is monitored with a sampling frequency of 5 Hz (one measure every 0.2 second). (**B**) Maximum (expiration) and minimum (inspiration) values are automatically detected, cycle-to cycle, by automatic computer analysis. The maximum corresponds to the end-expiratory alveolar concentration of eCO and the minimum corresponds to the CO concentration in ambient air or medical gas. To calculate the actual eCO after correction of ambient CO, the software automatically subtracts, cycle by cycle, the minimum from the maximum.

This laser spectroscopy technique and its potential interest in clinical implementation have been described in details in previous publications^8–11^. Briefly, laser spectroscopy measurements of very low gas concentrations (less than 1 ppm for endogenous expired CO for a non-smoker patient) require a large light absorption path. As some other spectroscopy techniques, OF-CEAS exploits a resonant optical cavity that allows an effective optical absorption path length of several kilometers while the cavity is only one meter long (folded in two arms) and its volume is about 20 cm^3^. This allows very sensitive measurements with compact instruments and a small sampling volume. Additionally, OF-CEAS provides absolute concentration measurements of sufficient accuracy without any periodic calibration with certified gas mixtures. In contrast to the somewhat complex physics underlying OF-CEAS, its optical layout consists of few basic optical elements allowing for a compact and robust device. In this study we use an OF-CEAS analyzer (ProCeas) commercialized by the AP2Ecompany (Aix-en-Provence, France). This instrument, including electronics for laser control and data acquisition, fits inside a 19 inches rack and is perfectly adapted to a medical environment (Fig. 1). The ProCeas continuously measures CO concentration by extracting a flow of 0.15 L/min from the airway circuit of the anesthesiology station. Measurements are performed at a reduced pressure of 140 mbar in the optical cavity. The CO detection limit is about 1 ppb for an acquisition time of 0.2 second. (Fig. 2A). The response time is limited to 1 second by the gas exchange time in the cavity. For each eCO measurement, a zone of interest of about one minute of the cycle-to-cycle recorded curve was selected (Fig. 2B). An automatic computer analysis detected the maximum and minimum values of each cycle and calculate the difference, for each respiratory cycle, between inhaled and exhaled CO. This provides the actual amount of endogenous CO produced by the organism, regardless of CO levels in ambient air or in medical gases supplied to the anesthesiology station. Another research group, using a close laser spectroscopy technic, coupled with eCO2 expirograms measured by capnography and laser spectroscopy, confirmed the true real-time detection of this technic^17^. It as to be noted that eCO exhalation profiles have a slightly different shape than the eCO2 profiles (due to its local production by airway epithelium) therefore showing a dependence on exhalation flow rate and breath-holding time.

### eCO measurement with an electrochemical sensor (MicroCO)

All along the experiment we also measured eCO with a standard electrochemical fuel cell handheld sensor (MicroCO; VYaire Fig. 1). This instrument is commonly used in smoking cessation clinical programs or for detection of CO poisoning. The comparison between the two devices was not the main goal of our study as, by construction, the two devices display very different characteristic and sensitivities. Indeed, the MicroCO resolution is 1 ppm with a response time below 15 second. Comparison with the MicroCO is useful to illustrate the wide difference between today available medical devices and the accuracy and speed attainable with a ProCeas laser spectrometer. The MicroCO device was carefully calibrated before the experiments using a calibration gas containing 20 ppm of CO (from VYaire). For measurements, a sample of exhaled air was taken during mid-outbreath using a 60 mL syringe connected to a three-way valve placed in the airway circuit. The air in the syringe was then slowly expelled into the MicroCO. Each MicroCO measurement was performed three times, then results were averaged.

### Statistical analysis

The sample-size estimation (NQuery Advisor 7.0 software; Statistical Solutions Ltd, Boston MA, USA) was based on data from previous publications^15,16,18^ with the aim to detect a difference of 5 ppm in eCO measured by ProCeas between FiO_2_ 21% and FiO_2_ 100% (patient being his own control) with a power of 90% and a 2-tailed significance level of 0.05. For the comparison of eCO values taken at different FiO_2_, we used paired ***t*** test with Bonferroni correction for five repeated tests. Consequently, only *p* values < 0.01 were considered as significant. eCO results are finally presented as mean ± standard error (m ± S.E.). Correlation and reliability between ProCeas and MicroCO devices were tested by linear regression analysis and Blant and Altman plots.

### Ethics approval and consent to participate

The study protocol was approved by the French “Comity for Protection of People” (CPP of region South East 5) (registration number 2008-A00273-52). Only adults volunteers participated to the experiment. Written consent was obtained from every subjects.

## Results

30 healthy participants (29.7 ± 7.2 years old with a sex ratio of 53.7% of women) were included in this experiment. 19 subjects were non-smokers whereas 11 subjects admitted smoking habits between 1 and 20 pack-years (mean: 8.6 ± 1.8 pack-years). The time between the last cigarette and the moment of eCO measurement was also variable among the smokers (from 2 hours to 24 hours). CO concentration gas in the airway circuit was monitored in real time during all the 30 minutes breathing by the ProCeas analyzer. Measurements during inhalation phases allow to deduce the CO background in inhaled air that is cycle by cycle subtracted to the exhaled CO concentration values, as described above in the methods section. For accurate measurements monitoring the inhaled CO concentration is required as it varies at each FiO_2_ step: from a few ppb at FiO2 100% (pure medical oxygen) to a hundred of ppb at FiO_2_ 21% (pure medical air) and has daily variations of hundreds ppb in ambient air (typically around 500 ppb). MicroCO was not sensitive enough to detect any CO in ambient air, neither in medical gases. eCO values measured while subjects breathed ambient air or medical air (FiO_2_ 21%) were approximately similar (mean 5.225 ± 0.624 ppm) regardless of the moment when high FiO_2_ values were applied. Breathing high FiO_2_ dramatically increased eCO (Table 1).

**Table 1:** Exhaled Carbon Monoxide values measured by the ProCeas in healthy volunteers (smokers and non-smokers together).

	Ambient air	Medical air	Medical gas mixture	Medical pure oxygen	Medical air
Time from beginning of experiment	5 min.	10 min.	15 min.	20 min.	25 min.
FiO_2_	21%	21%	50%	100%	21%
**Exhaled CO** (ppm)	4.98 ± 1.06	5.20 ± 1.08	9.30 ± 1.82 *	20.51 ± 4.08 **	5.50 ± 1.14 *

**p < 0.001 Exhaled CO with FiO_2_ 100% *versus* every other situation.

*p < 0.01 Exhaled CO with FiO_2_ 50% *versus* ambient air or medical air (FiO_2_ 21%).

Comparisons by paired ***t*** test, for n = 30.

**Figure 3:**
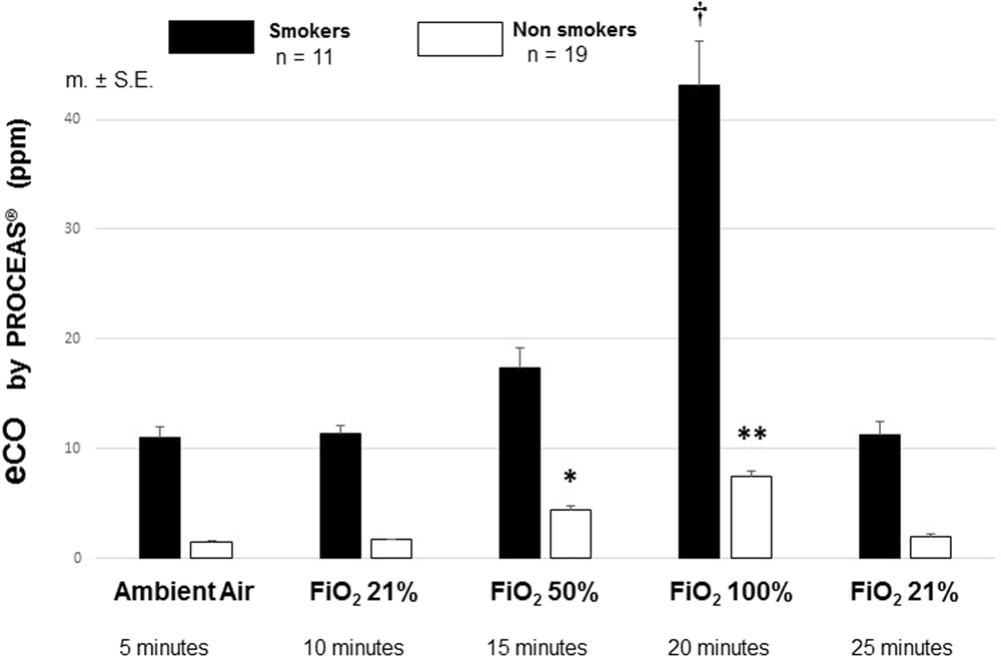
eCO measured by ProCeas in smokers (n = 11) and non-smoker (n = 19) subjects Values are given from the five minute duration of the steady-state after FiO_2_ changes. ^†^*p* < 0.001 eCO with FiO_2_ 100% *versus* every other situations in smokers. ^**^p < 0.001 eCO with FiO_2_ 100% *versus* every other situations in non-smoker subjects. ^*^p < 0.01 eCO with FiO_2_ 50% *versus* ambient air or medical air (FiO_2_ 21%) in non-smoker subjects. Comparisons by paired ***t*** test.

Subjects with smoking habits typically exhaled five times more eCO than non-smokers, regardless of FiO_2_ (Fig. 3). However, whatever the initial eCO values in ambient air or medical air, applying FiO_2_ 100% enhances eCO levels by a factor of four in smokers as well as in non smokers (Fig. 3). When FiO_2_ was switched back to 21%, eCO returned in a few minutes to the initial value measured before FiO_2_ elevation.

**Figure 4:**
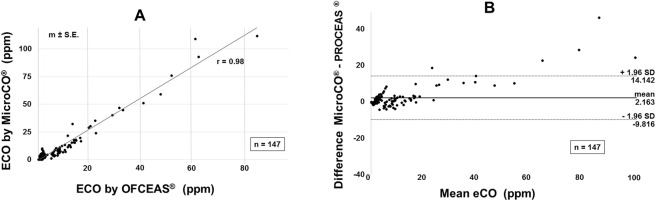
(**A,B**) Comparison of ProCeas and MicroCO measurements. (**A**) Correlation scatter plot; correlation represents the relationship between dataset; correlation coefficient is given as **r**. (**B**) Bland-Altman plots; eCO are given in ppm; vertical axis shows the eCO difference between the compared devices; mean eCO on the horizontal axis refers to the average of the eCO values measured by both devices.

ProCeas values correlated well with MicroCO measurements (correlation coefficient r = 0,98) (Fig. 4A). However Bland – Altman analysis showed a mean bias of 2.2 ± 6.1 ppm with underestimation by MicroCO for low concentrations (MicroCO detection limit is 1 ppm) and overestimation by MicroCO for high CO levels (Fig. 4B).

## Discussion

The main results of our study is that inhalation of 100% FiO_2_ increased eCO levels by a factor of four in every subjects (smokers and non smokers). Moreover the laser spectrometry analyzer ProCeas allows a precise and reliable cycle to cycle monitoring of endogenous exhaled CO. It measures accurately eCO elevations induced by FiO_2_ variations in healthy volunteers.

### Comparison of different intruments

To our knowledge, this is the first study with a laser spectroscopy based analyzer, monitoring one-line, cycle-to-cycle, eCO changes due to FiO_2_ variations in healthy volunteers. Different instruments have been used to measure eCO concentration. Gas chromatography or mass spectrometry analyzers, do not allow real time analysis and are too heavy and bulky for a convenient utilization in medical environment. Thus, until now, electrochemical devices were most frequently used in clinical practice.

#### Electrochemical sensors

The detection thresholds of these sensors are generally above 1 ppm. Measuring actual alveolar eCO concentrations under various FiO_2_ is challenging with electrochemical devices. In volunteers this can be obtained from a single breath at forced expiration^14^. In mechanically ventilated patients, obtaining end-tidal gas samples requires to stop the ventilation in end-inspiration phase, clamp and disconnect the endotracheal tube from the ventilator, then released the clamp so that all gas from the lungs is exhaled passively through the CO monitor to residual functional capacity^18^. Other authors collected exhaled breath at the outlet of the ventilator and either measured averaged eCO concentrations^15^, or picked-up highest eCO (as end-tidal values) among all collected data^16^. Compared to these methods, our one-line, cycle-to-cycle monitoring with the ProCeas, allows 5 measurements per second (5 Hz acquisition rate) and resolves each respiratory cycle. In our data, eCO level was lowest (1.71 ± 0.09 ppm) in non-smoker subjects at 21% FIO_2_. Such concentrations are close to the detection limit of the MicroCO. The range of eCO concentrations we measured at various FiO_2_ was roughly consistent with values found in other publications^14–16,18^. However, in our data, eCO measured by ProCeas almost quadrupled between 21% and 100% FiO_2_ whatever group being considered (smokers or non-smokers). This increase is greater than in other studies. It is also grater than our data with MicroCO which only detects a 2-fold eCO increase in non-smoker subjects breathing pure oxygen. It may be attributed to teh high sensitivity and the better resolution of the respiratory cycle of our measurements, that allows to accurately measure end-tidal values and also to subtract cycle by cycle the inhaled level of CO to estimate correctly endogenous CO production.

#### Laser spectrometers

Over the two last decades, optical laser spectrometers started being used for eCO measurement^11^. These devices are almost maintenance free and can operate continuously for long time periods. In 2000 Zegdi *et al*.^15^ published the first study using an infrared CO analyzer (CO 2000, Sérès, La Duranne, France) to measure eCO variations due to FiO_2_ elevation, in mechanically ventilated critically ill patients. This study displayed very low eCO values compare to our data and to other publications^14,16,18^. The explanation is probably that owing to the long response time of the CO 2000 device (70 s), only averaged eCO concentrations were measured rather than alveolar (end-tidal) concentrations. These measurements could not be performed on-line but exhaled breath had to be collected in bags placed at the outlet of the ventilator before analysis.

Other laboratory prototypes have been developed using various laser spectroscopy techniques to measure CO in exhaled breath. A laser spectrometer exploiting resonant cavity with a technique very close to the OF-CEAS (named cavity ring-down spectroscopy), demonstrates similarly low detection limit (1ppb in 1 second),on-line as well, but with a larger gas flow (0.7 L/min) extracted from the gas line for the laser analyzer^19^ So far, this research group validated the accuracy of their eCO measurements in few subjects and various situations: one smoker *versus* two non-smokers^19^, ten healthy volunteers practicing exercise^20^. However due to the use of a bulky laser(CO laser), unlike the ProCEAS, this system is a laboratory analyzer and is not transportable in medical environments. Compact analyzers adapted for eCO detection have been recently developed, based on the use of a multipass cell to enhance the light absorption path rather than a resonant optical cavity as used in OF-CEAS. Contrary to cavity enhanced techniques, the use of a multipass cell requires regular calibration (such as the commercial CO 2000 device used by Zegdi *et al*., calibrated every week). Despite the lower absorption path of a multipass cell, the use of new semi-conductor lasers (QCL and ICL in the mid infrared range where CO absorption may be stronger) allows the development of compact and sensitive analyzers. A detection limit as low as 7 ppb for an integration time of 1 second was achieved by Pakmanesh *et al*.^21^. Here, as for the ProCEAS, the response time is limited by the gas exchange time in the cell. The small multipass cell volume (47 mL) and the reduced pressure (20mbar) allows a gas exchange time of 1.7 s, that could be even reduced below 1 second by increasing the flow from 33 mL/min (value reported in^21^) to 100 mL/min. In comparison, the ProCeas operates at a sampling flow of 150 mL/min, flow that can be extracted from a ventilator gas line. More recently, Ghorbani *et al*.^22^ developed an instrument for eCO measurement that allows to resolve breath-cycle with a lower detection limit (~3 ppb in 1 second). With this device, different exhalation profile shapes are studied and a mathematical model is proposed to differentiate the CO gas exchange parameters between airways and alveoli. However, to reach such precision and resolution levels, this device needs to sample the gas with a flow of 3 L/min that has to be subtracted from the airway circuit^17^. Such flows represents almost the totality of normal human breath (tidal volume) at rest and is incompatible with an airway ventilator system as used in the work reported here.

### Tobacco and eCO

In our data, any subject having recently smoked showed higher eCO levels than non-smokers. This effect is well known and is used to control actual tobacco consumption in clinical programs for smoke cessation^23^.

### Oxygen and eCO

We found a systematic rise in eCO while increasing FiO_2_. eCO quadrupled between 21% and 100% FiO_2_ whatever was basal eCO level of each subject (smokers or non-smokers). eCO returned to basal values after FiO_2_ was switched back from 100% to 21%. This phenomenon is classically described in several publications^14–16,18^ within a broad spectrum of eCO values at comparable FiO_2_.

Schober *et al*.^18^ using an electrochemical sensor, measured eCO variation due to oxygen variation in volunteers. eCO values increased from 10.7 ± 5.9 ppm (21% FiO_2_) to 16.0 ± 6.0 ppm (100% FiO_2_). They also measured eCO in patients undergoing surgery, before and after pre-oxygenation for anesthesia, and after oro-tracheal intubation. Similarly to volunteers, in eCO increased from 7.1 ± 6.1 to 16.4 ± 8.6 ppm after 10 min pre-oxygenation with pure oxygen. Oro-tracheal intubation enhances this increased eCO up to 26.1 ± 13.1 ppm.

The same research group performed the same kind of measurements, just before anaesthesia, in 19 patients scheduled for cardiac surgery. Oxygen inhalation resulted in an increase in eCO levels from 8.6 ± 4.9 to 16.7 ± 9.4 ppm^14^. Adachi *et al*.^16^ studied (with an electrochemical sensor) the effect of FiO_2_ variations on exhaled CO, in thirty patients under general anaesthesia who underwent elective operations. They, all the same, found an eCO increase with high FiO_2_ (from 3.35 ± 0.62 ppm in basal value up to 7.57 ± 1.49 ppm under 100% FiO_2_). Zegdi *et al*.^15^ using an infrared CO analyzer, measured eCO variations due to FiO_2_ elevation, in nine mechanically ventilated critically ill patients. These authors described also a rise in eCO with pure oxygen (from 0.63 ± 0.13 ppm in basal value up to 1.54 ± 0.16 ppm under 100% FiO_2_)

In our study, the amplitude of the eCO increase we measured (4 fold increase) is larger than those described in all these previous publications.

Various clinical situations and possible lack of precision of instruments may explain the discrepancies. Indeed most of these studies employed electrochemical devices. Nonetheless, a marked elevation of eCO with increasing FiO_2_ is systematically found in the literature. At 100% FiO_2_ all studies showed an eCO increase by a factor 2 or 3 compared to basal levels. However frequently, basal values, in those series, are higher than found in our volunteers. Most of those previous publications concerned critically ill patients, or undergoing surgery, under mechanical ventilation. In Zegdi *et al*. study^15^ patients were all in intensive care unit and their “basal status” was a mechanical ventilation with 50% FiO_2_. This may perhaps explain the smaller difference (compare to ours) in eCO increase between their basal status and pure oxygen administration. Our healthy subjects (even if including non-symptomatic smokers) had probably less airways inflammation than those patients. Because eCO is elevated in many inflammatory situations, this could explain why basal eCO levels were higher in other series than in our study.

### Mechanisms of eCO increase under high FiO_2_

eCO elevation while increasing FiO_2_ may be due to a displacement of pre-existing CO from its hemoglobin bound. It could also originate as new CO production by hemoxygenase HO-1 induction. The few previous clinical studies investigating eCO changes while breathing oxygen-enriched air are in favor of the theory of CO displacement from hemoglobin. In Zegdi *et al*. study^15^ eCO rose markedly to a transient peak before returning to baseline values after seven hours of 100% FiO_2_. Schober *et al*.^14^ measured arterial oxygen tension (PaO2) and arterial carboxy-hemoglobin levels (CO-Hb), in mechanically ventilated patients. They showed a correlation between PaO2 and eCO levels which is in favor of the displacement of CO from hemoglobin bond. However, they failed to find a correlation between eCO concentrations and arterial CO-Hb levels. Adachi *et al*.^16^ have shown a decrease of blood CO-Hb concentrations over time, correlated to a return of eCO to the baseline (and even lower the baseline level) after four hours of ventilation at 75% FiO_2_. This findings argues in favor the CO displacement hypothesis. However, deleterious effects of high oxygen concentrations are known and hyperoxia is used to induce an experimental acute lung injury in several animal models^24^. Hyperoxia may provoke an over-expression of HO-1 that in turn brings about an overproduction of carbon monoxide^25^. Possibly this mechanism might account for a part of the eCO we measured in our subjects but we have not measured HO-1 in our healthy volunteers. As our subjects were only exposed to 100% FiO_2_ for a very short period (five minutes) we think that inflammatory reactions due to hyperoxia probably did not have sufficient time to occur nor did the production of CO from the degradation of the heme molecules. Therefore we hypothesis that our eCO increase under pure oxygen is mostly due to CO displacement from hemoglobin.

### Limits of the study

The carbon monoxide content in ambient air was not taken into account when recording MicroCO values, however this device was not sensitive enough to detect any CO in ambient air or in medical gases. No blood sample was collected during our experiments on healthy volunteers. Measurement of inducible hemeoxygenase (HO-1), carboxyhemoglobin (Hb-CO) or pro-inflammatory cytokines could have been of interest. However, the main goal of the study was to test the accuracy of the ProCeas in clinical setting rather than questioning the physiological CO production pathways. The study was conducted in healthy volunteers and not on critically ill patients whose profile of endogenous CO production is probably different from physiological pathways, due to inflammation and others pathological mechanisms.

### Future applications

CO is increasingly being accepted as a cytoprotective and homeostatic molecule with important signaling capabilities in physiological and pathophysiological situations^1,26^. Being able to monitor on line and precisely the endogenous production of CO could be of high interest in several clinical situations such as those concerning airways inflammation (COPD, Asthma, Cystic Fibrosis, lung transplantation). Some research teams also use eCO levels to estimate accurately red blood cell lifespan and study several forms of anemia^27^. As another application, the precision of the OF-CEAS technique allows to selectively measure lung CO production in an experimental *ex vivo* pig lung perfusion (EVLP) model in order to develop noninvasive identification of the most IR injured lungs^10^. One of our ongoing study is currently evaluating eCO measurements in human EVLP procedures in a clinical lung transplantation program.

## Conclusion

The ProCeas instrument, based on the OF-CEAS technique, demonstrated its suitability in clinical setting for on-line cycle-to-cycle monitoring of endogenous exhaled CO. In an operating room environment, ProCeas measured a four fold increase in eCO in healthy volunteers (smokers and non-smokers) breathing pure oxygen compared to air. This amplitude of eCO increase between 21% and 100% FiO_2_ is greater than those described in previous publications. We hypothesize that the precision of measurements and a better respiratory cycle resolution of ProCeas explains these results.

## Data Availability

The datasets used and analysed during the current study are available from the corresponding author on reasonable request.
